# Evaluating biomarkers for contrast-induced nephropathy following coronary interventions: an umbrella review on meta-analyses

**DOI:** 10.1186/s40001-024-01782-y

**Published:** 2024-04-01

**Authors:** Abinash Mahapatro, Sara Nobakht, Sindu Mukesh, Amir Ali Daryagasht, Aishwarya Reddy Korsapati, Shika M Jain, Saman Soltani Moghadam, Rozhin Moosavi, Mona Javid, Soheil Hassanipour, Shrinidhi Vilas Prabhu, Mohammad-Hossein Keivanlou, Ehsan Amini-Salehi, Sandeep S. Nayak

**Affiliations:** 1 Hi-Tech Medical College and Hospital, Rourkela, Odisha India; 2 Guilan University of Medical Sciences, Rasht, Iran; 3 Liaquat University of Medical and Health Sciences, Jamshoro, Pakistan; 4 University of Buckingham Medical School, Buckingham, UK; 5 MVJ Medical College and Research Hospital, Bengaluru, India; 6 Tehran University of Medical Sciences, Tehran, Iran; 7 Kasturba Medical College, Mangalore, India; 8 Department of Internal Medicine, Yale New Haven Health Bridgeport Hospital, Bridgeport CT, USA; 9 Gastrointestinal and Liver Diseases Research Center, Guilan University of Medical Sciences, Rasht, Iran; 10 Student Research Committee, School of Medicine, Guilan University of Medical Sciences, Rasht, Iran

**Keywords:** Contrast induced nephropathy, Biomarkers, Predicators, Cardiac catheterization, Meta-analysis, Umbrella review, Coronary angiography, Percutaneous coronary intervention

## Abstract

**Background:**

Contrast-induced nephropathy (CIN) is a form of acute kidney injury (AKI) occurring in patients undergoing cardiac catheterization, such as coronary angiography (CAG) or percutaneous coronary intervention (PCI). Although the conventional criterion for CIN detection involves a rise in creatinine levels within 72 h after contrast media injection, several limitations exist in this definition. Up to now, various meta-analyses have been undertaken to assess the accuracy of different biomarkers of CIN prediction. However, the existing body of research lacks a cohesive overview. To address this gap, a comprehensive umbrella review was necessary to consolidate and summarize the outcomes of prior meta-analyses. This umbrella study aimed to offer a current, evidence-based understanding of the prognostic value of biomarkers in predicting CIN.

**Methods:**

A systematic search of international databases, including PubMed, Scopus, and Web of Science, from inception to December 12, 2023, was conducted to identify meta-analyses assessing biomarkers for CIN prediction. Our own meta-analysis was performed by extracting data from the included studies. Sensitivity, specificity, positive likelihood ratio, and negative likelihood ratio were assessed using Meta-Disc and CMA softwares.

**Results:**

Twelve studies were ultimately included in the umbrella review. The results revealed that neutrophil gelatinase-associated lipocalin (NGAL) exhibited the highest area under the curve (AUC), followed by cystatin-C, urinary kidney injury molecule-1 (uKIM-1), and brain natriuretic peptide (BNP) with AUCs of 0.91, 0.89, 0.85, and 0.80, respectively. NGAL also demonstrated the highest positive likelihood ratio [effect size (ES): 6.02, 95% CI 3.86–9.40], followed by cystatin-C, uKIM-1, and BNP [ES: 4.35 (95% CI 2.85–6.65), 3.58 (95% CI 2.75–4.66), and 2.85 (95% CI 2.13–3.82), respectively]. uKIM-1 and cystatin-C had the lowest negative likelihood ratio, followed by NGAL and BNP [ES: 0.25 (95% CI 0.17–0.37), ES: 0.25 (95% CI 0.13–0.50), ES: 0.26 (95% CI 0.17–0.41), and ES: 0.39 (0.28–0.53) respectively]. NGAL emerged as the biomarker with the highest diagnostic odds ratio for CIN, followed by cystatin-C, uKIM-1, BNP, gamma-glutamyl transferase, hypoalbuminemia, contrast media volume to creatinine clearance ratio, preprocedural hyperglycemia, red cell distribution width (RDW), hyperuricemia, neutrophil-to-lymphocyte ratio, C-reactive protein (CRP), high-sensitivity CRP, and low hematocrit (*P* < 0.05).

**Conclusion:**

NGAL demonstrated superior diagnostic performance, exhibiting the highest AUC, positive likelihood ratio, and diagnostic odds ratio among biomarkers for CIN, followed by cystatin-C, and uKIM-1. These findings underscore the potential clinical utility of NGAL, cystatin-C and uKIM-1 in predicting and assessing CIN.

**Graphical Abstract:**

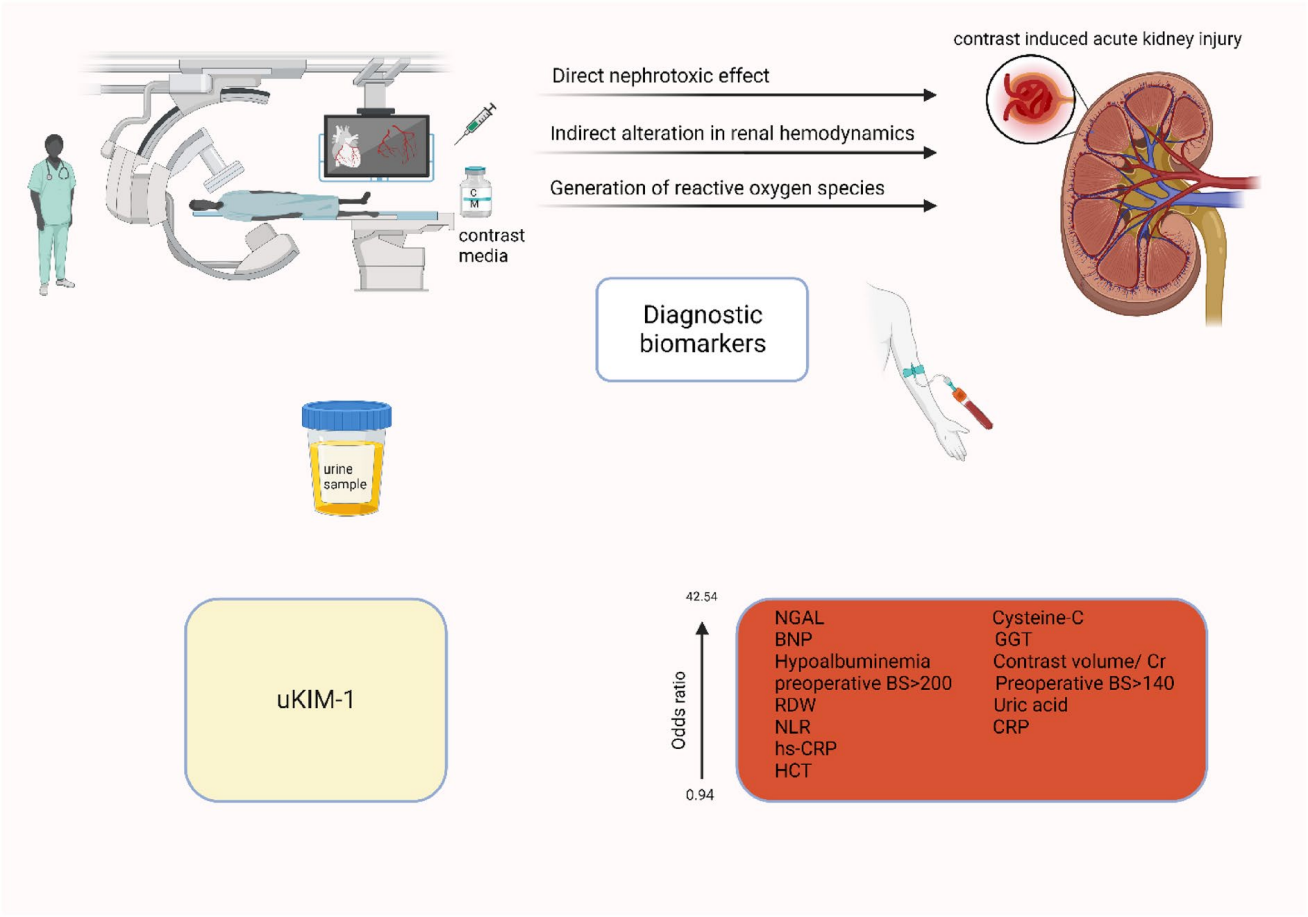

**Supplementary Information:**

The online version contains supplementary material available at 10.1186/s40001-024-01782-y.

## Introduction

Contrast-induced nephropathy (CIN), also referred to as contrast-induced acute kidney injury (CI-AKI), is a renal injury caused by the administration of radio-opaque contrast media (CM) into the vasculature, particularly in individuals who are susceptible to such adverse effects. The origins of CIN can be traced back to the 1950s when case reports documented instances of fatal acute renal failure subsequent to intravenous pyelography in patients with renal complications associated with multiple myeloma [1, 2].

Despite advancements in technology, CIN continues to be accountable for approximately one-third of all instances of hospital-acquired acute kidney injury (AKI) [3, 4]. Its prevalence ranges from 1 to 2% in the general population and can escalate to as much as 50% in high-risk subgroups undergoing procedures such as coronary angiography (CAG) or percutaneous coronary intervention (PCI) [5].

Nowadays CIN is defined as a 25% relative increase, or a 0.5 mg/dL (44 µmol/L) absolute increase, in serum creatinine (SCr) within 72 h of contrast exposure in the absence of alternative conditions [6]. It is reported, that renal impairment occurring up to 7 days after contrast delivery is classified as CIN if it cannot be attributed to any other potential cause of renal failure. Serum creatinine levels peak 2–5 days after contrast exposure and typically revert to baseline in 14 days [7].

In addition to the rise in serum creatinine, recent studies propose alternative biomarkers that could predict CIN. Numerous original studies and meta-analyses have delved into this area. For instance, a recent meta-analysis conducted by Javid et al. proposes that Gamma-glutamyl transferase (GGT) could serve as a reliable predictor of CIN. Meanwhile, Wu et al. suggest that inflammatory markers such as C-reactive protein (CRP) and neutrophil to lymphocyte ratio (NLR) might also be indicative of CIN [8, 9].

Until now, several meta-analyses have introduced a variety of biomarkers for predicting CIN after cardiac catheterization. Nevertheless, there exists a need for an umbrella review to comprehensively assess and summarize the outcomes of prior meta-analyses and scrutinize the quality of their findings. In this umbrella review, our objective was to appraise the biomarkers identified in previous meta-analyses as predictors of CIN. We intend to assess their robustness using power analysis to provide a thorough evaluation of their strength.

## Methods

Our umbrella review, a systematic review encompassing various meta-analyses, adhered to the guidelines outlined in the Cochrane Handbook for Systematic Reviews [10]. The presentation of results followed the Preferred Reporting Items for Systematic Reviews and Meta-Analyses (PRISMA) guidelines [11]. The study protocol has been preregistered in PROSPERO under the registration identifier CRD42023493911.

### Search strategy

To identify meta-analyses assessing predictors of CIN after cardiac catheterization, two independent researchers (A.M and S.N), designed a comprehensive search formula. This formula was applied across three international databases (PubMed, Scopus, and Web of Science) from their inception up to December 12, 2023. The search included keywords such as "Angiography," "Cardiac Catheterization," "Acute Kidney Injury," "Nephropathy," "Acute Renal Injuries," "Meta-Analysis," and "Systematic Review".

To ensure the precision of the search strategy, the expertise of two information specialists was enlisted. Additionally, a manual review of references from relevant studies was conducted. There was no language restriction. In instances of disagreement, resolution was achieved through the involvement of a third researcher (E.AS). The organization and management of identified studies were facilitated using EndNote X20. The detailed search formulas for each database are presented in Additional file 1: Table S1.

### Study selection and eligibility criteria

Two independent researchers (AM and SN) selected the studies, with any disparities being resolved through correspondence (EAS). The eligible meta-analyses adhered to specific criteria: the population included individuals who had undergone cardiac catheterization, encompassing procedures such as CAG or PCI. Furthermore, the studies were required to report CIN as an outcome of cardiac catheterization. Additionally, within this selected population, at least one biomarker had to be assessed as a predictor of CIN. Narrative reviews, original studies, commentaries, and editorials were excluded from the study.

### Quality assessment

The methodological quality of the included meta-analyses was evaluated using the AMSTAR2 checklist [12]. Two reviewers (A.M, and S.N) provided answers to 16 items on this checklist with the options "yes," "no," or "partial yes." Any disagreements were resolved by the third researcher (E.AS). Based on the checklist score, the studies were then divided into four groups: high quality, moderate quality, low quality, and critically low quality.

### Data extraction

The information extracted from the meta-analyses includes the first author's name, the publication year, the journal of publication, the study's country, the number of incorporated original studies, the total sample size, predictors of CIN, effect size and 95% confidence interval, heterogeneity for each outcome, and the funding source, and the checklist for evaluating the quality of the original studies. This information was transcribed into an Excel spreadsheet format.

To ensure that the dataset was complete, we contacted the corresponding and primary authors to address any missing information. Two researchers (A.M and S.N) worked together to carefully extract the data. If there were any disagreements, a third researcher was consulted to help resolve the issue.

### Statistical analyses

In the analysis of the current umbrella review, Comprehensive Meta-Analysis (CMA) version 4 was utilized. For the construction of receiver operating characteristic (ROC) charts pertaining to reported biomarkers, Meta-disc software version 1.4 was employed. For assessing summary receiver operating characteristics (SROC), Moses’ constant of linear model was utilized. Der Simonian-Laird model was used to calculate the effect sizes (ES) of sensitivity, specificity, positive like-hood ratio, and negative like-hood ratio.

The results of previous meta-analyses were summarized using the pooled results and 95% confidence intervals of the biomarkers. In instances where two or more meta-analyses evaluated a common biomarker, the selection criterion was based on the meta-analysis with the largest sample size. To ascertain the adequacy of sample sizes, power analyses were carried out for each outcome. Furthermore, the prediction intervals (PI) for each outcome were calculated using CMA software.

## Results

**Fig. 1:**
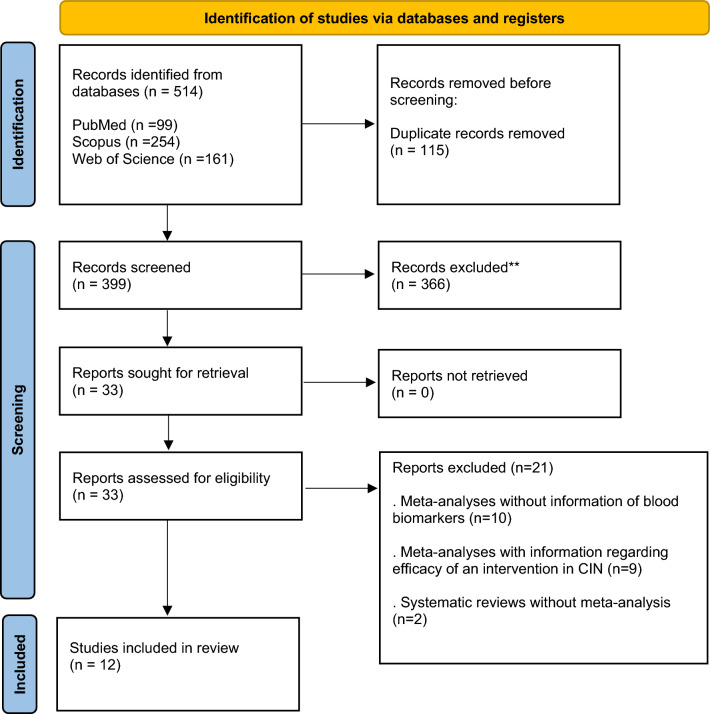
Study selection process

In the initial phase of the search, a total of 514 studies emerged, with 99 retrieved from PubMed, 254 from Scopus, and 161 from Web of Science. Following the elimination of 115 duplicates and screening of titles and abstracts in the remaining articles, 33 studies progressed to a thorough full-text assessment. Ultimately, after this evaluation, 12 studies were eligible to be included in the Umbrella review. The process of study selection is depicted in Fig. 1.

### Study characteristic

Among the 12 studies incorporated in this analysis, 10 studies originated from China [9, 13–21], with the remaining two studies from Iran [8] and the USA [20]. The publication timeline ranged from 2016 to 2023. The number of studies included and the total sample size exhibited variability, ranging from 4 to 26 studies and 946 to 29,454 participants, respectively.

A comprehensive evaluation of 16 markers, including albumin, blood urea nitrogen (BUN), C-reactive protein (CRP), highly sensitive C-reactive protein (hs-CRP), neutrophil-to-lymphocyte ratio (NLR), red cell distribution width (RDW), hematocrit (HCT), gamma-glutamyl transpeptidase (GGT), uric acid (UA), urinary kidney injury molecule-1 **(**uKIM-1), blood glucose, brain natriuretic peptide (BNP), cysteine C, neutrophil gelatinase-associated lipocalin (NGAL), contrast media volume to creatinine clearance ratio (V/cr), and platelet-to-lymphocyte ratio (PLR), was conducted by the meta-analyses included in this review.

Quality assessments were executed using various checklists. Five meta-analyses employed the Newcastle Ottawa scale (NOS) checklist [9, 13, 15, 18, 21], four utilized the quality assessment of diagnostic accuracy studies QUADS 2 checklist [14, 17, 19, 22], one used the QUADS checklist [16], one utilized Joanna Briggs Institute (JBI) [8], and one employed both the NOS and Cochrane Collaboration tool [20]. Funding information revealed that four studies received financial support [16, 17, 19, 22], while the remainder did not have any financial backing [8, 9, 13–15, 18, 20, 21]. As per the AMSTAR2 checklist, six studies were rated as critically low quality [15–17, 19, 20, 22], four as low quality [8, 9, 13, 21], and two as high quality [14, 18]. Only one study reported a previously registered protocol in PROSPERO [14], with the remaining 11 studies lacking information on a registered protocol [8, 9, 13, 15–22]. Detailed information regarding the included studies is summarized in the Table 1. Detailed information regarding the quality of included studies is presented in Additional file 1: Table S2.

**Table 1:** Characteristics of included studies

First author name, year of publication	Journal	Searched databases	Date of search	Number of included studies	Total sample size	Reported biomarkers	Checklist used for quality assessment of included original studies	Funding status	Previous registered protocol	Software of analysis	Model of analysis	Amstar 2 score
Zuo [13]	International Journal of Cardiology	PubMed, Embase, Cochrane Library, and CBM (Chinese Biomedical Literature database)	April 18, 2016	18	13,084	UA	NOS	No	No	STATA	Random-effects model	Low quality
Javid [8]	Annals of medicine and surgery	PubMed, Scopus, and Web of Science	November 2022	4	1346	GGT	JBI	No	No	CMA	Random-effects model	Low quality
Wu [9]	Angiology	PubMed, EMBASE, Google Scholar, Clinical Trials, and Science Direct	June 3, 2020	26	29,454	CRP, hsCRP, NLR, PLR, HCT, RDW	NOS	No	No	Revman	Random effect model for heterogenic and fix effect model for non-heterogenic studies	Low quality
Wu [14]	Medicine	PubMed, Embase, Cochrane Central Register of Controlled Trials Library, and Web of Science	March 9, 2022	12	7789	BNP	QUADAS-2	No	PROSPERO (CRD42022318275)	STATA, Review Manager, and MetaDisc	Random effects model	High quality
Zhang [15]	Angiology	PubMed, EMBASE, Google Scholar, Clinical Trials, and ScienceDirect	July 13, 2018	18	16,171	Alb, Uric acid, BUN	NOS	No	No	Revman	Random effect model for heterogenic and fix effect model for non-heterogenic studies	Critically Low-quality
Wang [16]	Canadian Journal of Cardiology	PubMed, MEDLINE, EMBASE, Web of Science, ClinicalTrials.gov, Cochrane Library, and Google Scholar	November 2014	14	1520	NGAL	QUADAS	Yes	No	STATA	Random effect model for heterogenic and fix effect model for non-heterogenic studies	Critically Low quality
Li [17]	Journal of Interventional Cardiology	Medline, Embase, ClinicalTrials.gov, Cochrane Library database, and the China National Knowledge Infrastructure (CNKI)	November 31, 2019	9	946	uKIM-1	QUADAS-2	Yes	No	STATA	Bivariate mixed-effects regression	Critically Low-quality
Nie [18]	Angiology	PubMed, Embase, and the Cochrane library	November, 2020	6	16,899	Contrast media volume to creatinine clearance ratio	NOS	No	No	STATA	Random effect model	High Quality
Li [22]	Journal of Interventional Cardiology	PubMed, EMBASE, and the Cochrane Central Register of Controlled Trials Library	April 2020	9	2832	BNP	QUADAS-2	Yes	No	STATA	Bivariate random-effects regression	Critically Low quality
Kewcharoen [20]	Cardiovascular Revascularization Medicine	MEDLINE and EMBASE	January 2020	8	10,991	Preprocedural blood glucose	NOS and Cochrane Collaboration tool	No	No	STATA	Random effects model	Critically Low quality
Chen [19]	Toxicology Letters	PubMed, MEDLINE, and Embase	March 2018	10	2554	Cystatin C	QUADAS-2	Yes	No	STATA	Bivariate meta-analysis	Critically Low quality
Jiang [21]	Medicine	PUBMED, EMBASE, and Web of Science	September 15, 2018	6	10,452	PLR	NOS	No	No	STATA and Revman	Random effect model for heterogenic and fix effect model for non-heterogenic studies	Low quality

Abbreviations: NOS: Newcastle Ottawa scale, JBI: Joanna Briggs Institute, QUADS: Quality Assessment of Diagnostic Accuracy Studies, UA: Uric Acid, GGT: Gamma-glutamyl transpeptidase, CRP: C-Reactive Protein, hsCRP: Highly Sensitive C-Reactive Protein, NLR: Neutrophil-to-lymphocyte ratio, RDW: red cell distribution width, PLR: Platelet-to-Lymphocyte Ratio, HCT: Hematocrit, Alb: Albumin, BUN: Blood urea nitrogen, NGAL: Neutrophil gelatinase-associated lipocalin, uKIM-1: Urinary kidney injury molecule-1, BNP: Brain natriuretic peptide

### Diagnostic accuracy of biomarkers

Five meta-analyses assessed the diagnostic accuracy of four biomarkers by reporting the number of true positive, false positive, true negative, and false negative cases within original studies (Cystatin-C, BNP, uKIM-1, NGAL) [14, 16, 17, 19, 22].

**Fig. 2:**
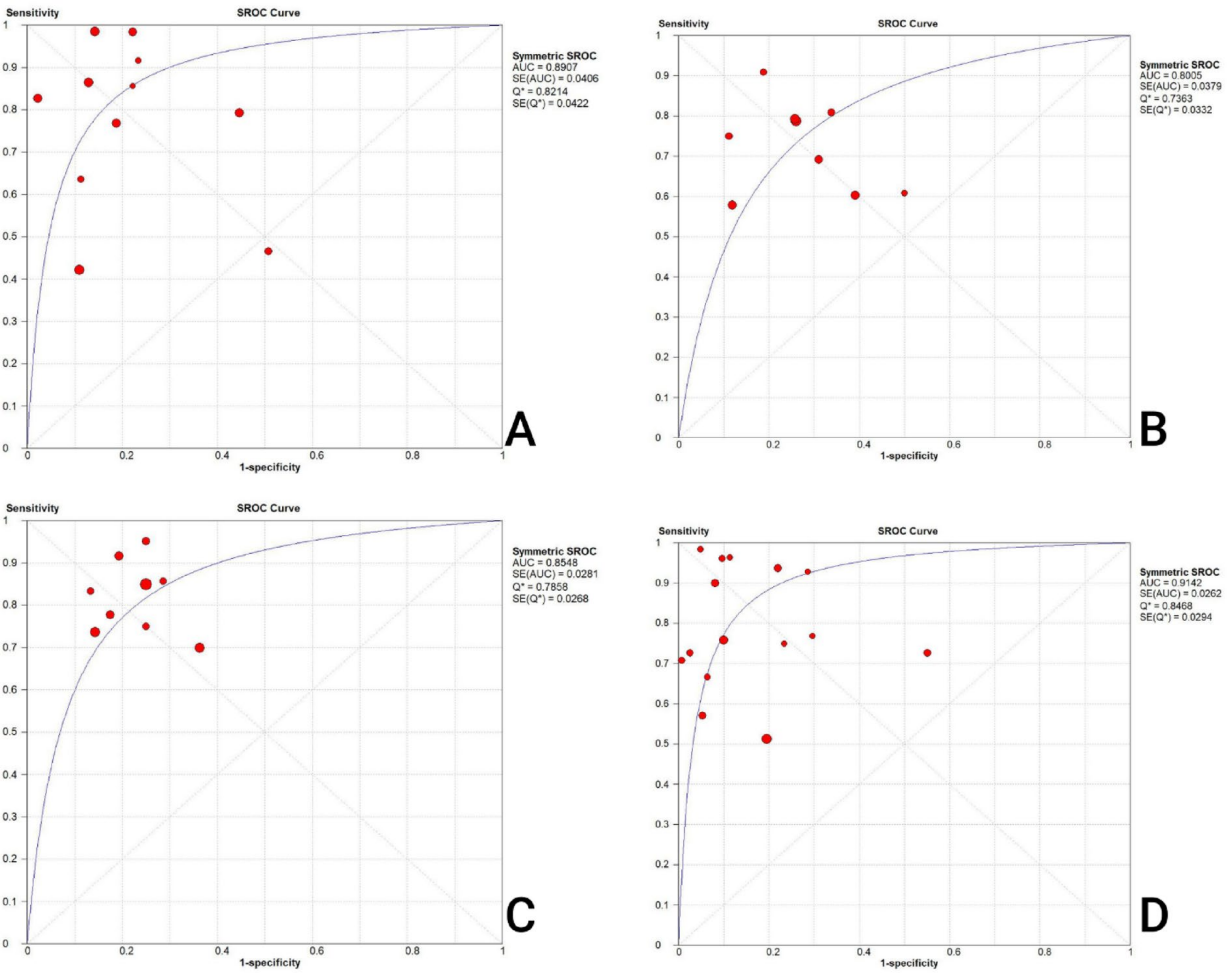
Summary receiver operating characteristics (SROC). **A** SROC for Cystatin-C, **B** SROC for BNP, **C** SROC for uKIM-1, **D** SROC for NGAL

Based on the results of the included meta-analyses, the pooled sensitivity, specificity, positive like-hood ratio, and negative hood ratio of serum/plasma Cystatin-C for predicting CIN were 0.74 and 0.81, 4.35 and 0.25 respectively. The sensitivity, specificity positive likelihood ratio, and negative likelihood ratio were accompanied by significant heterogeneity (88.7% and 96.4%, 93.7% and 90.3%, respectively). The area under curve (AUC) for Cystatin C was stated as 0.90 (Table 2 and Fig. 2).

**Table 2:** Diagnostic accuracy of biomarkers

Biomarker	Specificity	Sensitivity	Positive like hood ratio	Negative like hood ratio	AUC	Heterogeneity of sensitivity	Heterogeneity of specificity	Heterogeneity of Positive like hood ratio	Heterogeneity of Negative like hood ratio	Q
Cystatin-C	0.81 (0.79–0.82)	0.74 (0.69–0.79)	4.35 (2.85- 6.65)	0.25 (0.13–0.50)	0.89	88.7%, *P* < 0.01	96.4%, *P* < 0.01	93.7%, *P* < 0.01	90.3%, *P* < 0.01	0.82
BNP	0.73 (0.71–0.75)	0.72 (0.67–0.77)	2.85 (2.13–3.82)	0.39 (0.28–0.53)	0.80	56.1%, *P* = 0.01	94.0%, *P* < 0.01	89.1%, *P* < 0.01	70.6%, *P* < 0.01	0.73
uKIM-1	0.76 (0.73–0.79)	0.84 (0.78–0.88)	3.58 (2.75–4.66)	0.25 (0.17–0.37)	0.85	42.4%, *P* = 0.08	64.0%, *P* < 0.01	51.1% *P* = 0.03	20.3% *P* = 0.26	0.78
NGAL	0.85 (0.83–0.86)	0.78 (0.73–0.83)	6.02 (3.86–9.40)	0.26 (0.17–0.41)	0.91	73.1%, *P* < 0.01	91.6%, *P* < 0.01	85.0%, *P* < 0.01	65.5%, *P* < 0.01	0.84

NGAL: Neutrophil gelatinase-associated lipocalin, uKIM-1: Urinary kidney injury molecule-1, BNP: Brain natriuretic peptide

Based on the results obtained from the meta-analyses, the sensitivity, specificity, positive likelihood ratio, and negative likelihood ratio of BNP for predicting CIN were 0.72, 0.73, 2.85, and 0.39, respectively. Notably, the reported values for sensitivity, specificity, positive likelihood ratio, and negative likelihood ratio were associated with varying degrees of heterogeneity, with I^2^ statistics of 56.1%, 94.0%, 89.1%, and 70.6%, respectively. Additionally, the AUC for BNP, as determined was calculated at 0.80 (Table 2 and Fig. 2).

The outcomes from the meta-analyses revealed that for uKIM-1, the sensitivity, specificity, positive likelihood ratio, and negative likelihood ratio for predicting CIN were 0.84, 0.76, 3.58, and 0.25, respectively. The heterogeneity of sensitivity, specificity, positive likelihood ratio, and negative likelihood ratio was 42.4%, 64.0%, 51.1%, and 20.3%, respectively. Additionally, the AUC for uKIM-1 was found to be 0.85 (Table 2 and Fig. 2).

The outcomes of the meta-analyses revealed that for NGAL in predicting CIN, the sensitivity, specificity, positive likelihood ratio, and negative likelihood ratio were 0.78, 0.85, 6.02, and 0.26, respectively. The heterogeneity for sensitivity, specificity, positive likelihood ratio, and negative likelihood ratio was 73.1%, 91.6%, 85.0%, and 65.5%, respectively. Furthermore, the AUC for NGAL was 0.91 (Table 2 and Fig. 2).

### Diagnostic adds ratio of biomarkers

**Fig. 3:**
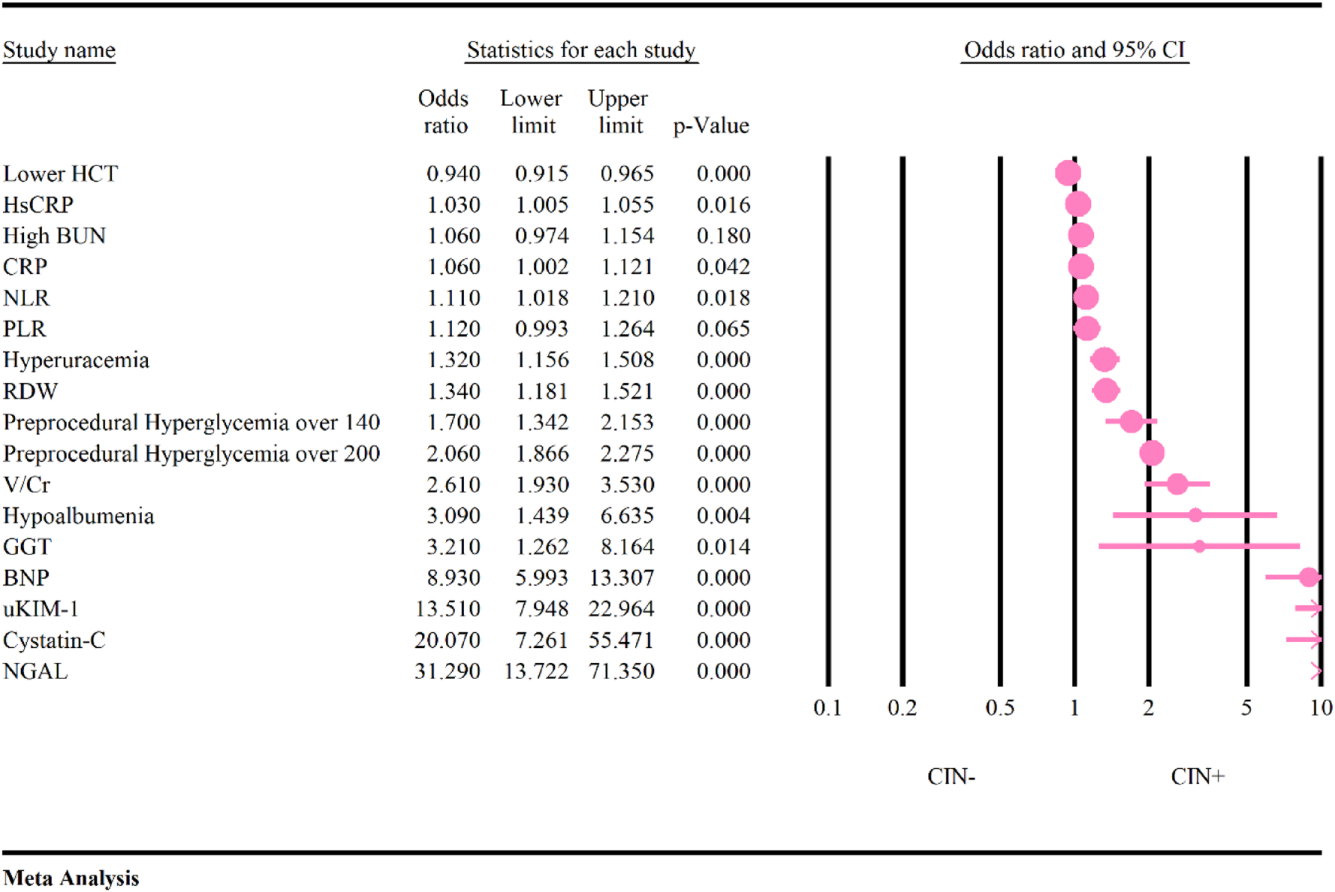
Diagnostic odds ratio of biomarkers

**Fig. 4:**
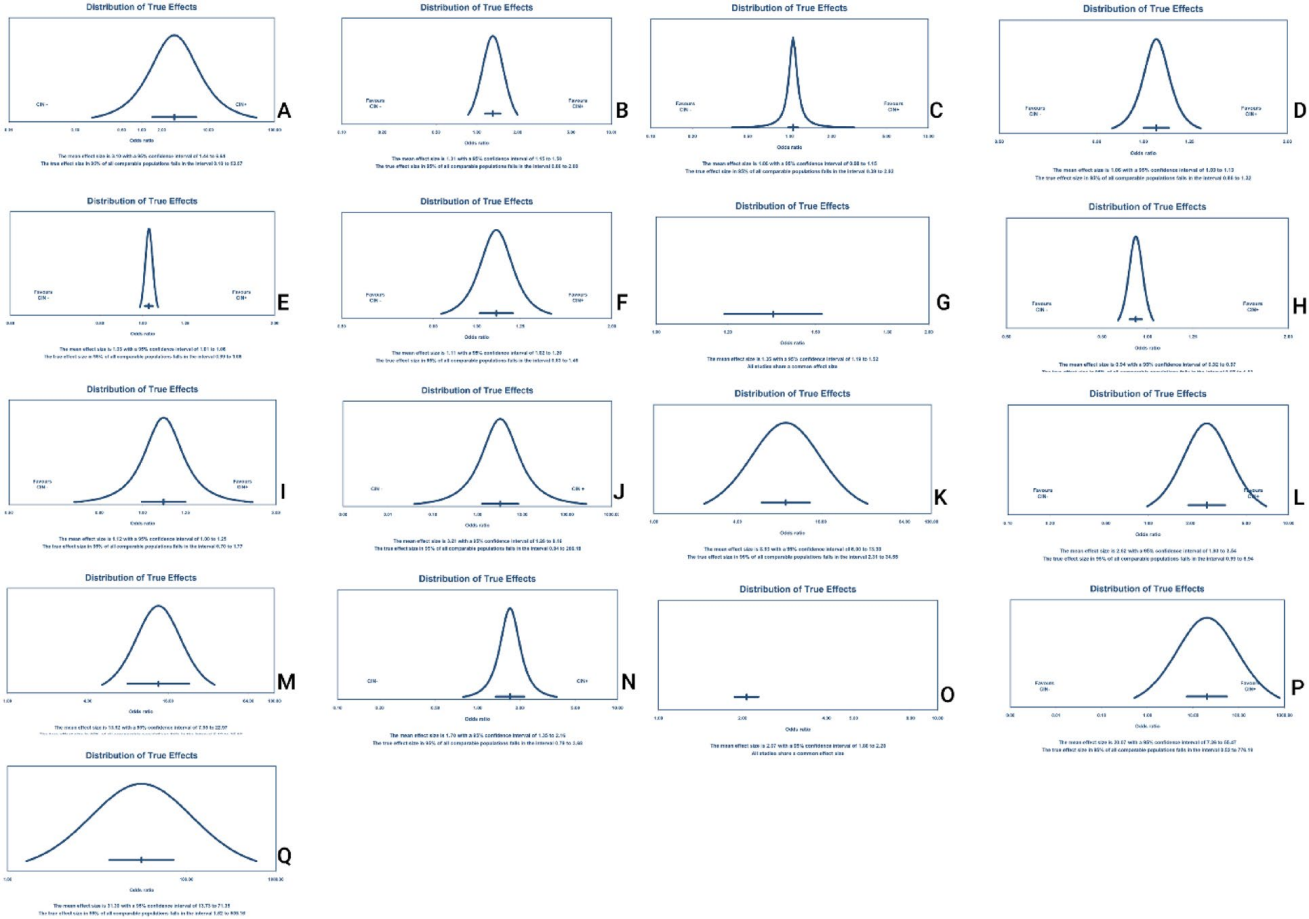
Results of prediction interval of the biomarkers. **A** Hypoalbuminemia, **B** Hyperuricemia, **C** BUN, **D** CRP, **E** hs-CRP, **F** NLR, **G** RDW, **H** Lower HCT, **I** PLR, **J** GGT, **K** BNP, **L** V/Cr, **M** uKIM-1, **N** Preprocedural hyperglycemia over 140, **O** Preprocedural hyperglycemia over 200, **P** Cystatin-C, **Q** NGAL

In the preceding meta-analyses, comprehensive evaluations of the diagnostic odds ratios for a total of 16 biomarkers were conducted. The correlation of two biomarkers, including BUN and PLR, with CIN, was found to be statistically insignificant (*P* > 0.05) (Fig. 3 and Table 3). Conversely, the remaining biomarkers exhibited a significant association with the occurrence of CIN. Notably, NGAL emerged as the biomarker with the highest diagnostic odds ratio for predicting CIN (OR = 31.29, 95% CI 13.72–71.35, *P* < 0.01, PI: 1.61–605.16), followed by Cystatin C and uKIM-1 (OR = 20.07, 95% CI 7.26–55.47, *P* < 0.01, PI: 0.51–776.18 and OR = 13.51, 95% CI 7.94–22.96, *P* < 0.01, P: 5.12–35.66, respectively) (Figs. 3 and 4, Table 3). In contrast, hs-CRP (OR = 1.03, 95% CI 1.01–1.05, *P* = 0.01, PI:0.98–1.08) demonstrated the lowest diagnostic odds ratio, followed by CRP and HCT (OR = 1.06, 95% CI 1.00 -1.12, *P* = 0.02, PI: 0.86–1.31 and OR = 0.94, 95% CI 0.91–0.96, *P* < 0.01, PI: 0.86–1.03, respectively) (Figs. 3 and 4, Table 3).

**Table 3:** Results of meta-analysis, heterogeneity, publication bias, power analysis and prediction interval of biomarkers

Variable	Odds ratio	95% CI, *P* value	Heterogeneity	*P* vale of publication bias based on eagers regression test	Power analysis	Prediction interval
Hypoalbuminemia	3.09	1.43–6.63, *P* < 0.01	90%, *P* < 0.01	*P* = 0.17	1-b = 1	0.17–53.56
Hyperuricemia	1.32	1.15–1.50, *P* < 0.01	88%, *P* < 0.01	*P* < 0.01	1-b = 0.99	0.86–2.00
BUN	1.06	0.97–1.15, *P* = 0.18	88%, *P* < 0.01	*P* = 0.52	1-b = 0.05	0.38–2.91
CRP	1.06	1.00–1.12, *P* = 0.04	98%, *P* < 0.01	*P* = 0.36	1-b = 0.15	0.86–1.31
HS-CRP	1.03	1.01–1.05, *P* = 0.01	50%, *P* = 0.04	*P* < 0.01	1-b = 0.12	0.98–1.08
NLR	1.10	1.01–1.20, *P* = 0.01	78%, *P* < 0.01	*P* = 0.26	1-b = 0.46	0.83–1.46
RDW	1.34	1.18–1.52, *P* < 0.01	0%, *P* = 0.73	*P* = 0.30	1-b = 0.70	1.18–1.52
Lower HCT	0.94	0.91–0.97, *P* < 0.01	69%, *P* < 0.01	*P* = 0.74	1-b = 0.33	0.86–1.03
PLR	1.11	0.99–1.24, *P* = 0.06	87%, *P* < 0.01	*P* = 0.09	1-b = 0.43	0.70–1.77
GGT	3.21	1.26–8.15, *P* = 0.01	91%, *P* < 0.01	*P* = 0.06	1-b = 1	0.03–266.17
BNP	8.93	5.99–13.30, *P* < 0.01	69%, *P* < 0.01	*P* = 0.06	1-b = 1	2.30–34.55
V/CR	2.61	1.93–3.53, *P* < 0.01	76%, *P* < 0.01	*P* < 0.01	1-b = 1	0.98–6.94
UKIM-1	13.51	7.95–22.97, *P* < 0.01	14%, *P* = 0.31	*P* = 0.01	1-b = 1	5.12–35.66
Preprocedural hyperglycemia over 140	1.70	1.34–2.15, *P* < 0.01	26%, *P* = 0.25	*P* = 0.45	1-b = 1	0.78–3.68
Preprocedural hyperglycemia over 200	2.06	1.87–2.28, *P* < 0.01	0%, *P* = 0.90	*P* = 0.30	1-b = 1	1.87–2.28
Cystatin-C	20.07	7.26–55.46, *P* < 0.01	88%, *P* < 0.01	*P* = 0.11	1-b = 1	0.51–776.18
NGAL	31.29	13.72–71. 34, *P* < 0.01	73%, *P* < 0.01	*P* < 0.01	1-b = 1	1.61–605.16

Gamma-glutamyl transpeptidase, CRP: C-Reactive Protein, hsCRP: Highly Sensitive C-Reactive Protein, NLR: Neutrophil-to-lymphocyte ratio, RDW: red cell distribution width, PLR: Platelet-to-Lymphocyte Ratio, HCT: Hematocrit, BUN: Blood urea nitrogen, NGAL: Neutrophil gelatinase-associated lipocalin, uKIM-1: Urinary kidney injury molecule-1, BNP: Brain natriuretic peptide, V/Cr: Contrast volume to creatinine clearance

### Results power analysis

**Fig. 5:**
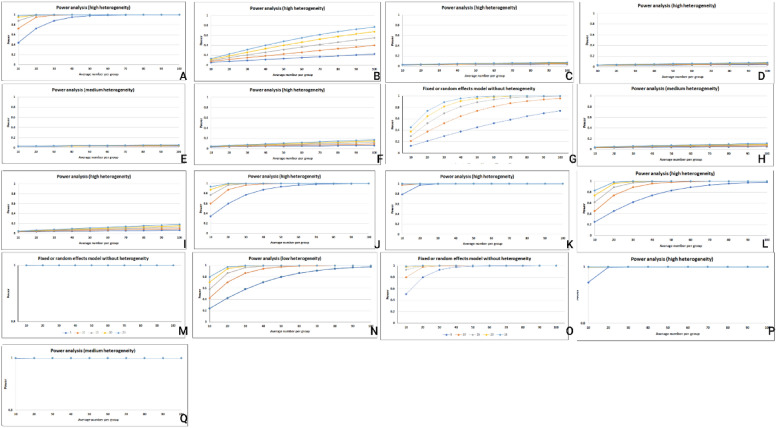
Results of power analysis of the biomarkers. **A** Hypoalbuminemia, **B** Hyperuricemia, **C** BUN, **D** CRP, **E** hs-CRP, **F** NLR, **G** RDW, **H** Lower HCT, **I** PLR, **J** GGT, **K** BNP, **L** V/Cr, **M** uKIM-1, **N** Preprocedural hyperglycemia over 140, **O** Preprocedural hyperglycemia over 200, **P** Cystatin-C, **Q** NGAL

Based on the results of meta-analyses, the following biomarkers were associated with low statistical power: BUN (1-β = 0.05), CRP (1-β = 0.15), Hs-CRP (1-β = 0.12), NLR (1-β = 0.46), Lower HCT (1-β = 0.33), and PLR (1-β = 0.43). In contrast, the prognostic value of the remaining biomarkers was associated with high statistical power (Fig. 5 and Table 3).

### GRADE assessment

The epidemiological strength of outcomes was rigorously assessed using the GRADE criteria. Results indicated one high-quality outcome (BNP), two with moderate quality (Cystatin-C and GGT), nine with low quality (NGAL, uKIM-1, albumin, RDW, PLR, NLR, CRP, BUN, HCT), and five with very low quality (V/CR, preprocedural hyperglycemia over 200, preprocedural hyperglycemia over 140, uric acid, HS-CRP). The detailed information regarding GRADE criteria is presented in Table 4.

**Table 4:** GRADE assessment of the biomarkers

Quality assessment							Quality
No of studies	Design	Risk of bias	Inconsistency	Indirectness	Imprecision	Other considerations	
NGAL							
14	Observational studies	Serious	No serious inconsistency	No serious indirectness	No serious imprecision	Reporting bias very strong association	⊕⊕〇〇 low
Cystatin-C							
10	Observational studies	Serious	No serious inconsistency	No serious indirectness	No serious imprecision	Very strong association	⊕⊕⊕〇 moderate
uKIM-1							
9	Observational studies	Serious	No serious inconsistency	No serious indirectness	No serious imprecision	Reporting bias very strong association	⊕⊕〇〇 low
BNP							
12	Observational studies	No serious risk of bias	No serious inconsistency	No serious indirectness	No serious imprecision	Very strong association	⊕⊕⊕⊕ high
GGT							
4	Observational studies	No serious risk of bias	No serious inconsistency	No serious indirectness	No serious imprecision	Strong association	⊕⊕⊕〇 moderate
Albumin							
5	Observational studies	No serious risk of bias	No serious inconsistency	No serious indirectness	No serious imprecision	Reporting bias strong association	⊕⊕〇〇 low
V/CR							
6	Observational studies	Serious	No serious inconsistency	No serious indirectness	No serious imprecision	Reporting bias strong association	⊕〇〇〇 very low
Preprocedural hyperglycemia over 200							
7	Observational studies	Serious	Serious	No serious indirectness	No serious imprecision	Strong association	⊕〇〇〇 very low
Preprocedural hyperglycemia over 140							
4	Observational studies	Serious	Serious	No serious indirectness	No serious imprecision	None	⊕〇〇〇 very low
RDW							
5	Observational studies	No serious risk of bias	No serious inconsistency	No serious indirectness	No serious imprecision	None	⊕⊕〇〇 low
Uric acid							
18	Observational studies	No serious risk of bias	No serious inconsistency	No serious indirectness	No serious imprecision	Reporting bias	⊕〇〇〇 very low
PLR							
4	Observational studies	No serious risk of bias	No serious inconsistency	No serious indirectness	No serious imprecision	None	⊕⊕〇〇 low
NLR							
5	Observational studies	No serious risk of bias	No serious inconsistency	No serious indirectness	No serious imprecision	None	⊕⊕〇〇 low
HS-CRP							
9	Observational studies	No serious risk of bias	No serious inconsistency	No serious indirectness	No serious imprecision	Reporting bias	⊕〇〇〇 very low
CRP							
5	Observational studies	No serious risk of bias	No serious inconsistency	No serious indirectness	No serious imprecision	None	⊕⊕〇〇 low
BUN							
3	Observational studies	No serious risk of bias	No serious inconsistency	No serious indirectness	No serious imprecision	None	⊕⊕〇〇 low
HCT							
8	Observational studies	No serious risk of bias	No serious inconsistency	No serious indirectness	No serious imprecision	None	⊕⊕〇〇 low

## Discussion

Contrast-induced acute kidney injury (CI-AKI) frequently occurs following percutaneous coronary intervention (PCI) or coronary angiography (CAG). It leads to extended hospital stays, elevated healthcare costs, and, in certain instances, heightened cardiovascular and renal morbidity and mortality [23]. Despite its substantial impact, there are limited established strategies for preventing CI-AKI [24]. Recognizing patients at an early stage who are at a high risk of developing CI-AKI is crucial. While serum creatinine (SCr) concentration is widely acknowledged as the conventional clinical measure for identifying and characterizing CI-AKI, it has several limitations. Notably, it is influenced by factors such as muscle metabolism diet, gender, and hydration, and its alteration rate following the initial injury is slow [25]. These characteristics render SCr an unreliable and temporally inadequate biomarker for diagnosing CI-AKI. Consequently, its utilization may compromise the effectiveness of treatments and corrective interventions.

Another risk assessment criterion for CIN is the Mehran risk score, designed to stratify the risk among patients [26]. While the Mehran risk score has demonstrated efficacy in numerous studies with robust power, certain variables included in the assessment, such as the volume of contrast medium and the use of an intra-aortic balloon pump, remain uncertain before the procedure. This uncertainty poses limitations on the practical clinical application of the Mehran risk score. Similar challenges and limitations are observed in other predictive models for CIN [27, 28].

The principal aim of the present study was to assess the diagnostic value of biomarkers in predicting CIN by evaluating and summarizing the results of previous meta-analyses. In addition, an assessment of the quality and power of these meta-analyses was conducted, enabling the strength of the results to be appraised and gaps in the existing literature to be identified.

Among the studies included in our analysis, five meta-analyses reported the raw figures for false positive, false negative, true positive, and true negative cases related to four biomarkers (Cystatin-C, BNP, uKIM-1, NGAL) [14, 16, 17, 19, 22]. Specifically, Wu et al. [14] and Li et al. [22] conducted meta-analyses on BNP, and due to the larger overall sample size in Wu et al. (7789 vs. 2832), we opted to report the results from their study in the current umbrella review.

In our examination, NGAL demonstrated the highest AUC and positive likelihood ratio among the variables, followed by Cystatin-C, uKIM-1, and BNP. Conversely, uKIM-1 exhibited the least negative likelihood ratio, followed by Cystatin-C, NGAL, and BNP.

Upon calculating and comparing the odds ratios of the sixteen biomarkers, NGAL emerged as the biomarker with the highest diagnostic odds ratio for CIN, followed by Cystatin-C and uKIM-1. Conversely, hs-CRP exhibited the lowest diagnostic odds ratio, with CRP and HCT ranking just above.

In the meta-analysis conducted by Wang et al. [16] additional investigations explored the predictive value of NGAL. Their findings suggested that evaluating NGAL within four hours after CM injection yielded superior results compared to assessments conducted beyond this timeframe, though not statistically significant. Notably, the study observed no significant difference between serum and urine NGAL levels in predicting CIN. Furthermore, the prognostic efficacy of NGAL levels in predicting CI-AKI according to a non-traditional definition was notably higher than its predictive accuracy for CI-AKI according to the traditional definition, although this was also not statistically significant.

It is noteworthy to mention that NGAL presents certain limitations in predicting CIN. Specifically, NGAL may not serve as a reliable biomarker in patients with chronic kidney disease (CKD). For example, a prospective, randomized controlled trial conducted by Ribitsch et al. which included 617 patients with CKD, demonstrated the limited efficacy of urinary NGAL in predicting CIN after angiography [29]. Moreover, the presence of urinary protein may interfere with the accuracy of NGAL measurements, further complicating its use as a biomarker for predicting CIN [30]. Furthermore, NGAL measurements can be influenced by various other factors. In a prospective observational study conducted by Kumar et al. it was discovered that age, estimated Glomerular Filtration Rate (eGFR), hemoglobin levels, and the volume of contrast used are significantly correlated with NGAL levels [31]. Sepsis and inflammation represent other critical underlying conditions that can influence NGAL levels [32, 33]. Recent studies have introduced additional variables, such as Insulin-Like Growth Factor Binding Protein 7 (IGFBP7) and Tissue Inhibitor of Metalloproteinases (TIMP), as potential biomarkers for predicting AKI in patients with sepsis [34]. Additionally, the accuracy of NGAL as a biomarker can be compromised by the presence of comorbid diseases. This further underscores the complexity of relying on NGAL for predicting CIN [35].

**Fig. 6:**
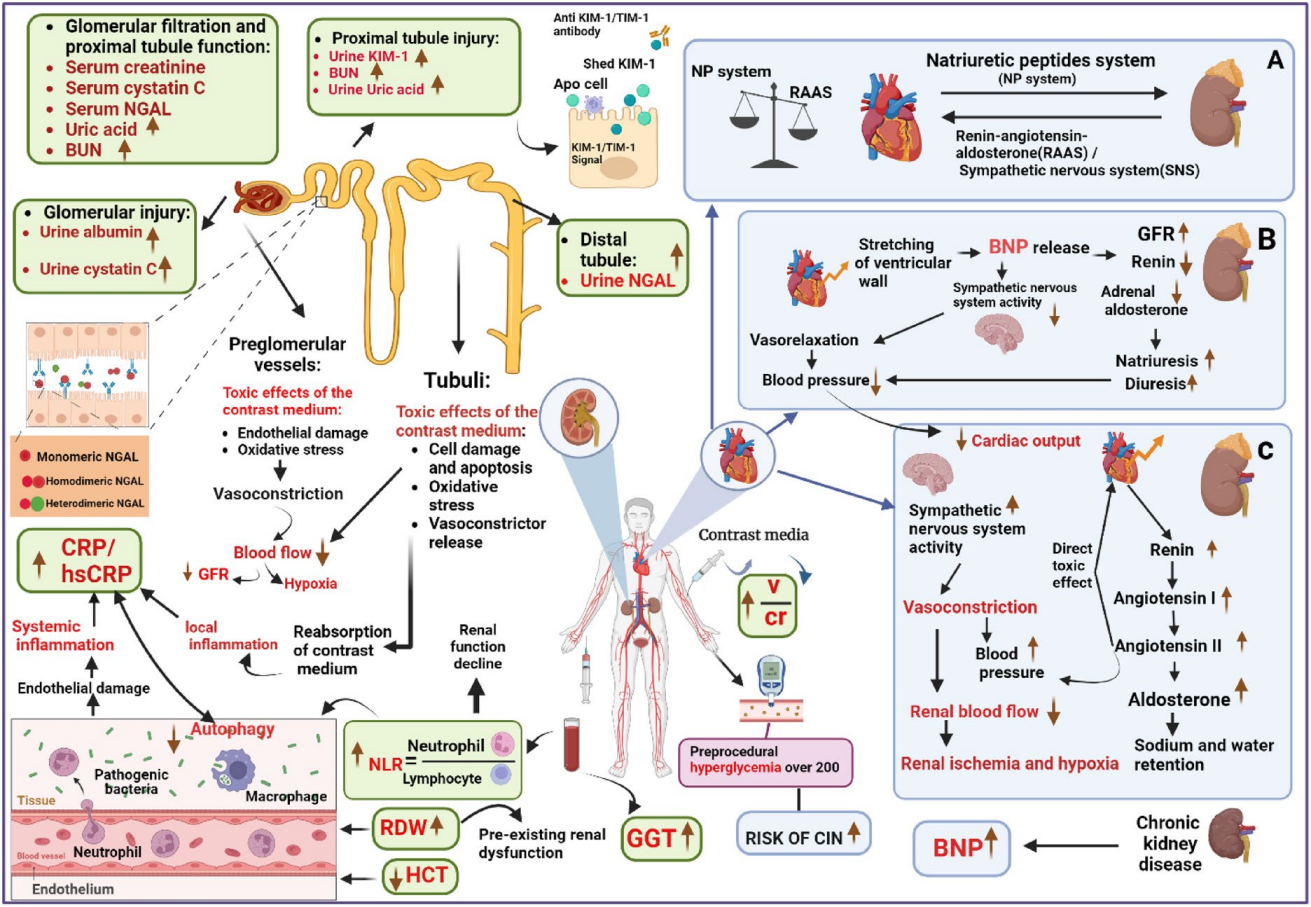
Biomarkers for prediction of contrast-induced nephropathy

NGAL is a factor that serves multiple roles in renal epithelial cells, including promoting growth, differentiation, and structural organization. It also plays an important role in preventing cell death and preserving the structure of the renal tubules, which helps to ensure the integrity of the kidneys [36, 37]. Studies have shown that exogenous NGAL can have a protective effect on the kidneys in mouse models of renal ischemia–reperfusion injury [36]. However, CM can be harmful to the kidneys by causing an increase in tubular osmolarity and impairing intracellular transport and energy metabolism in the tubular epithelial cells [38]. As a result, NGAL level increases rapidly after exposure to CM and is considered a critical factor in predicting the development of CI-AKI [16] (Fig. 6).

In the meta-analysis conducted by Chen et al. [19], the predictive strength of Cystatin-C for CIN was subjected to further scrutiny. Their findings revealed that measuring Cystatin-C after 24 h following CM injection demonstrated a higher diagnostic odds ratio compared to assessments conducted within the initial 24 h post-CM injection. The results of our study align with these findings, positioning Cystatin-C as one of the top three biomarkers for predicting CIN. Importantly, Cystatin-C offers distinct advantages over serum creatinine, as it is less influenced by patient characteristics such as muscle mass or diet. This characteristic enhances its reliability as a biomarker compared to creatinine [39, 40]. Furthermore, Cystatin-C boasts a shorter half-life than creatinine and exhibits an earlier increase in AKI. Consequently, measuring Cystatin-C enables the earlier diagnosis of CIN compared to measuring creatinine [41]. These attributes collectively underscore the potential clinical utility and superiority of Cystatin-C in the early detection and prediction of CIN. Cystatin C is generated by nucleated cells and undergoes free filtration through the glomerulus. It is reabsorbed but not secreted by the renal tubules [42–44]. Due to such physiological attributes, cystatin C has demonstrated superiority to SCr in identifying slight reductions in glomerular filtration rate especially in the setting of renal injuries [45–49] (Fig. 6).

The outcomes of the meta-analysis conducted by Li et al. [17] underscored that uKIM-1 exhibits enhanced diagnostic accuracy when measured 24 h after CM injection as opposed to assessments made within the initial 24 h post-CM injection. This aligns with findings from other studies, which have consistently reported the favorable diagnostic accuracy of uKIM-1 in the context of AKI. A comprehensive meta-analysis involving 11 studies and 2979 subjects yielded a diagnostic odds ratio of 17.43, with sensitivity and specificity for uKIM-1 in AKI reported at 74% and 86%, respectively [50]. Another meta-analysis by Ho et al. highlighted the robust diagnostic accuracy of uKIM-1 in the context of cardiac surgery-induced AKI, revealing AUC values of 0.68 for intraoperative and 0.72 for postoperative AKI [51]. Functionally, KIM-1 serves as a phosphatidylserine receptor expressed in epithelial cells. In kidney stress conditions, uKIM-1 plays a crucial role in identifying and phagocytizing dead cells [52]. While uKIM-1 is not detectable in urine under normal circumstances, its levels significantly increase in AKI due to proximal tubule damage [53, 54] (Fig. 6).

The result of the current umbrella review underscored the significance of hepatorenal status as a prognostic factor for CIN. Notably, individuals with hypoalbuminemia face an elevated risk of developing CIN. The connection between lower serum albumin levels upon admission and the occurrence of angiographic no-reflow following PCI has been established. This association not only exacerbates renal perfusion impairment but also increases the likelihood of CIN onset [55, 56]. Several other studies have identified hypoalbuminemia as a risk factor for AKI in diverse clinical settings, including liver transplantation, rhabdomyolysis, and cardiac surgery [56–60]. The potential mechanisms linking CIN and serum albumin levels revolve around oxidative stress, inflammation, and endothelial dysfunction, collectively predisposing individuals to CIN. Particularly, hypoalbuminemia may compromise the antioxidant defenses against heightened reactive oxygen species during renal ischemia. Consequently, this could intensify the vasoconstrictor effects of contrast media, ultimately leading to the induction of CIN [61] (Fig. 6).

Serum uric acid, a byproduct of purine metabolism, has garnered significant recognition for its role in diverse pathophysiological processes in recent years. Compelling evidence indicates that elevated levels of serum uric acid are linked to an increased risk of developing CKD [62, 63]. Beyond its direct impact on renal function, hyperuricemia is implicated in the development of CIN [63, 64] (Fig. 6).

Our investigation revealed that BNP serves as another predictor of CIN. Notably, BNP has been well-established as a biomarker for heart failure, as acknowledged in clinical guidelines [65, 66]. Moreover, a recent retrospective cohort study focusing on elderly individuals with chronic heart failure and a history of AKI found that a relative decline in serum BNP levels is associated with improved survival outcomes, potentially preventing the occurrence of AKI [67]. The complete understanding of the mechanisms through which BNP predicts CIN remains unclear. Various potential explanations have been suggested. Initially, BNP is produced in cardiomyocytes and is discharged into the bloodstream when there is stretching of the ventricular wall due to elevated pressure or increased volume. Elevated level of BNP is linked to a decrease in cardiac output, potentially impacting the hemodynamics of the renal artery. Secondly, BNP is considered as an indicator of the renin-angiotensin aldosterone system and the sympathetic nervous system, suggesting a potential increase in renal vascular resistance and a reduction in renal blood flow. This, in turn, could result in renal ischemia and hypoxia [68, 69]. Thirdly, patients with chronic kidney disease, even in the absence of cardiovascular abnormalities, exhibit heightened levels of BNP. Apart from a reduced extraction rate from the blood, elevated levels of BNP may signify a decline in functional renal mass and degradation of clearance receptors. Consequently, individuals with higher levels of BNP may be more susceptible to the nephrotoxic effects of contrast medium [70–72] (Fig. 6).

Based on the result of the current umbrella review, high CRP, and hsCRP are significantly associated with increased risk of CIN. CRP and hs-CRP are representative of systemic inflammation and systemic inflammation heightens the susceptibility of the kidneys to the local inflammatory responses triggered by the reabsorption of contrast medium [73]. Elevated CRP levels are additionally linked to endothelial injury and compromised vasodilation, potentially resulting in acute renal damage and the gradual decline of kidney function [74]. In addition, Animal studies further indicate that the downregulation of autophagy is linked to severe ischemia–reperfusion-induced AKI in mice that overexpress CRP, suggesting that CRP renders the kidney more vulnerable to ischemic/oxidative injury by suppressing autophagy flux [75]. Apart from PCI, the preoperative concentration of CRP is a prognostic indicator for the occurrence of postoperative AKI in patients undergoing coronary artery bypass grafting [76]. An increased level of hsCRP is associated with a heightened risk of AKI and the progression of chronic kidney disease after myocardial infarction, independent of the patient's initial renal status [77] (Fig. 6).

NLR offers a straightforward yet promising assessment of systemic inflammation and is employed as an indicator for cardiovascular diseases [78]. Its reliability as an inflammatory prognostic marker persists when considering coronary heart disease mortality, whether patients have ST-segment elevation myocardial infarction (STEMI), non-ST-segment elevation acute coronary syndrome, or peripheral artery disease [79–82]. In heart failure patients with reduced left ventricular ejection fraction, the NLR level was linked to the progression of renal dysfunction [83]. Additionally, the NLR was predictive of the deterioration of renal function in diabetic patients [84] (Fig. 6).

In our study, lower HCT was significantly associated with CIN. Another study involving 6773 patients revealed that a lower baseline HCT was an independent predictor of CIN, regardless of the presence of chronic kidney disease. The study observed a significantly elevated risk of CIN with each 3% reduction in baseline HCT [85]. The correlation between HCT and inflammation may be a contributing factor to these findings, as indicated by the previous study and our meta-analysis [9, 86] (Fig. 6).

The Cholesterol and Recurrent Events study highlighted that elevated RDW values may indicate an underlying inflammatory condition, playing a significant role in the development of CIN [87]. Moreover, abnormal RDW has been linked to preexisting impaired renal function and increased mortality in diverse clinical settings, including patients with acute myocardial infarction, patients experiencing hemodialysis, and recipients of kidney transplants [88–90] (Fig. 6).

### Strengths, limitations, and future suggestions

To our knowledge, this study represents a comprehensive umbrella review focusing on meta-analyses that investigate predictors of CIN in patients following CAG or PCI. Our comprehensive examination involved the evaluation of biomarkers identified in previous meta-analyses. Additionally, we meticulously assessed the quality of these meta-analyses, identifying gaps in the existing literature. Furthermore, we evaluated the sufficiency of the reported results through a rigorous power analysis, providing insights into the robustness of the evidence. Recognizing the potential impact of heterogeneity on the reported findings, we incorporated the calculation of prediction intervals to offer a more nuanced interpretation of the results.

Despite these methodological strengths, several limitations were encountered in the course of this study. Firstly, only five meta-analyses provided detailed information on the crude numbers of false positives, false negatives, true positives, and true negatives among patients. Consequently, our ability to assess metrics such as sensitivity, specificity, AUC, positive likelihood ratio, and negative likelihood ratio for the remaining biomarkers was restricted. Secondly, the power analysis underscored a limitation within the results, as evidenced by the lower power observed among the included biomarkers, BUN, CRP, Hs-CRP, NLR, Lower HCT, and PLR. This outcome warrants caution in interpreting the findings of the aforementioned biomarkers, given the associated low power attributed to these biomarkers due to the relatively small sample size. To enhance the robustness of conclusions drawn from such biomarkers, future investigations should prioritize larger sample sizes.

Furthermore, it is important to note that, with the exception of RDW, blood glucose, and uKIM-1, the remaining biomarkers exhibited significant heterogeneity. This heterogeneity may stem from diverse variables, including variations in the demographic characteristics of the study populations, discrepancies in the volume of contrast administered during CAG or PCI, variations in renal function among patients exposed to CM, differences in the types of CM employed across meta-analyses, and disparities in the definitions of CIN. These factors underscore the complexity of the relationships between biomarkers and clinical outcomes, necessitating a careful interpretation of the results. Future studies should consider addressing and controlling for these sources of heterogeneity to provide more accurate insights into the clinical implications of the examined biomarkers.

Furthermore, during our appraisal of the included studies' quality, a significant observation was the lack of study protocol registration in the majority of the included meta-analyses. Moreover, a substantial portion of these meta-analyses did not adequately address the potential impact of low-quality studies on their reported results. To enhance the robustness of future investigations, we recommend the incorporation of subgroup analyses based on the quality of the original studies.

Moreover, a noteworthy observation was the lack of information regarding the optimal timing for biomarker measurement in the majority of the included studies. We strongly encourage future research endeavors to assess the predictive value of biomarkers at various time points following CM injection. This exploration will contribute valuable insights into determining the most opportune moments for biomarker measurement, enhancing the precision and clinical applicability of these predictive markers for contrast-induced nephropathy.

### Supplementary Information

**Additional file 1. Table S1**: Search strategy for each international database. **Table S2**: Quality of included studies based on AMSTAR2 appraisal checklist.

## Data Availability

The datasets used and/or analyzed during the current study can be provided from the corresponding author on reasonable request.

## Abbreviations

AKI: Acute kidney injury
BNP: Brain natriuretic peptide
BUN: Blood urea nitrogen
CAG: Coronary angiography
CI-AKI: Contrast-induced acute Kidney Injury
CIN: Contrast-induced nephropathy
CM: Contrast media
CMA: Comprehensive meta-analysis
CRP: C-reactive protein
GGT: Gamma-glutamyl transpeptidase
HCT: Hematocrit
hs-CRP: Highly sensitive C-reactive protein
JBI: Joanna Briggs Institute
NLR: Neutrophil-to-lymphocyte ratio
NGAL: Neutrophil gelatinase-associated lipocalin
NOS: Newcastle Ottawa scale
PCI: Percutaneous coronary intervention
PI: Prediction interval
PLR: Platelet-to-lymphocyte ratio
QUADS: Quality assessment of diagnostic accuracy studies
RDW: Red cell distribution width
ROC: Receiver operating characteristic
SCr: Serum creatinine
SROC: Summary of receiver operating characteristic
STEMI: ST-segment elevation myocardial infarction
UA: Uric Acid
uKIM-1: Urinary kidney injury molecule-1
V/cr: Contrast media volume to creatinine clearance ratio
