# Supplementary material for: Evaluating biomarkers for contrast-induced nephropathy following coronary interventions: an umbrella review on meta-analyses
Source: Eur J Med Res. 2024 Apr 1;29:210. doi: 10.1186/s40001-024-01782-y (PMC10983745; doi:10.1186/s40001-024-01782-y)
Supplement: Supplementary file 1 — Additional file 1. Table S1: Search strategy for each international database. Table S2: Quality of included studies based on AMSTAR2 appraisal checklist. [file 40001_2024_1782_MOESM1_ESM.docx]

**Table S1: Search strategy for each international database**

| ISI | (("Cardiac Catheterizations" OR "Catheterizations, Cardiac" OR "Catheterization, Heart" OR "Heart Catheterization" OR "Catheterizations, Heart" OR "Heart Catheterizations" OR "Catheterization, Cardiac" OR "Angiography" OR "Angiographies" OR "Angiogram" OR "Angiograms" OR "Arteriography" OR "arteriographic" OR "Coronary Angiography" OR "Angiography, Coronary" OR "Angiographies, Coronary" OR "Coronary Angiographies" ) AND ("Acute Kidney Injuries" OR "Acute Kidney Injury" OR "Kidney Injuries, Acute" OR "Kidney Injury, Acute" OR "Acute Renal Injury" OR "Acute Renal Injuries" OR "Renal Injuries, Acute" OR "Renal Injury, Acute" OR "Renal Insufficiency, Acute" OR "Acute Renal Insufficiencies" OR "Renal Insufficiencies, Acute" OR "Acute Renal Insufficiency" OR "Kidney Insufficiency, Acute" OR "Acute Kidney Insufficiencies" OR "Kidney Insufficiencies, Acute" OR "Nephropathy") AND ("Contrast Media" OR "Media, Contrast" OR "Contrast Agent" OR "Agent, Contrast" OR "Contrast Materials" OR "Materials, Contrast" OR "Material, Contrast" OR "Radiocontrast Media" OR "Media, Radiocontrast" OR "Radiocontrast Agent" OR "Agent, Radiocontrast" OR "Radiocontrast Agents" OR "Agents, Radiocontrast" OR "Radiopaque Media" OR "Media,Radiopaque") AND ("Systematic Review" OR "Review, Systematic" OR "systematic rewiews" OR "metaanalysis" OR "Meta-Analysis" OR "metaanalysis" OR "meta analysis")) |
| --- | --- |
| PubMed | ((((("Angiography"[Mesh]) OR ("Coronary Angiography"[Mesh])) OR ("Cardiac Catheterization"[Mesh])) AND ("Contrast Media"[Mesh])) AND (("Acute Kidney Injury"[Mesh]) OR (nephropathy[Title/Abstract]))) AND (("Meta-Analysis" [Publication Type]) OR ("Systematic Review" [Publication Type])) |
| Scopus | TITLE-ABS-KEY ( "Cardiac Catheterizations" OR "Catheterizations, Cardiac" OR "Catheterization, Heart" OR "Heart Catheterization" OR "Catheterizations, Heart" OR "Heart Catheterizations" OR "Catheterization,Cardiac" OR "Angiography" OR "Angiographies" OR "Angiogram" OR "Angiograms" OR "Arteriography" OR "arteriographic" OR "Coronary Angiography" OR "Angiography, Coronary" OR "Angiographies, Coronary" OR "Coronary Angiographies" ) AND TITLE-ABS-KEY ( "Acute Kidney Injuries" OR "Acute Kidney Injury" OR "Kidney Injuries, Acute" OR "Kidney Injury, Acute" OR "Acute Renal Injury" OR "Acute Renal Injuries" OR "Renal Injuries, Acute" OR "Renal Injury, Acute" OR "Renal Insufficiency, Acute" OR "Acute Renal Insufficiencies" OR "Renal Insufficiencies, Acute" OR "Acute Renal Insufficiency" OR "Kidney Insufficiency, Acute" OR "Acute Kidney Insufficiencies" OR "Kidney Insufficiencies, Acute" OR "Nephropathy" ) AND TITLE-ABS-KEY ( "Contrast Media" OR "Media, Contrast" OR "Contrast Agent" OR "Agent, Contrast" OR "Contrast Materials" OR "Materials, Contrast" OR "Material, Contrast" OR "Radiocontrast Media" OR "Media, Radiocontrast" OR "Radiocontrast Agent" OR "Agent, Radiocontrast" OR "Radiocontrast Agents" OR "Agents, Radiocontrast" OR "Radiopaque Media" OR "Media,Radiopaque" ) AND TITLE-ABS-KEY ( "Systematic Review" OR "Review, Systematic" OR "systematic rewiews" OR "metaanalysis" OR "Meta-Analysis" OR "metaanalysis" OR "meta analysis" ) |

**Table S2: Quality of included studies based on AMSTAR2 appraisal checklist**

| Study, Year | Q1 | Q2 | Q3 | Q4 | Q5 | Q6 | Q7 | Q8 | Q9 | Q10 | Q11 | Q12 | Q13 | Q14 | Q16 | Q16 | Total quality |
| --- | --- | --- | --- | --- | --- | --- | --- | --- | --- | --- | --- | --- | --- | --- | --- | --- | --- |
| Zuo,2018 | Yes | No | Yes | Yes | Yes | Yes | Partial Yes | Yes | Yes | No | Yes | Yes | Yes | Yes | Yes | Yes | Low quality review |
| Javid, 2023 | Yes | No | Yes | Yes | Yes | Yes | Partial Yes | Yes | Yes | No | Yes | Yes | Yes | Yes | Yes | Yes | Low quality review |
| Wu,2022 | Yes | Yes | Yes | Yes | Yes | Yes | Partial Yes | Yes | Yes | No | Yes | Yes | Yes | Yes | Yes | Yes | High quality review |
| Wu.2021 | Yes | No | Yes | Yes | Yes | Yes | Partial Yes | Yes | Yes | No | Yes | Yes | Yes | Yes | Yes | Yes | Low quality review |
| Zhang,2019 | Yes | No | Yes | Yes | Yes | Yes | Partial Yes | Yes | Yes | No | Yes | Yes | Yes | Yes | No | Yes | Critically low quality review |
| Wang, 2016 | Yes | No | Yes | Yes | Yes | Yes | Partial Yes | Yes | Yes | No | Yes | No | No | Yes | Yes | Yes | Critically low quality review |
| Li, 2020 | Yes | No | Yes | Yes | Yes | Yes | Partial Yes | Yes | Yes | No | Yes | No | No | Yes | Yes | Yes | Critically low quality review |
| Nie, 2023 | Yes | No | Yes | Partial Yes | Yes | Yes | Partial Yes | Yes | Yes | No | Yes | No | No | Yes | Yes | Yes | Low quality review |
| Li,2020 | Yes | No | Yes | Yes | Yes | Yes | Partial Yes | Yes | Yes | No | Yes | No | No | Yes | Yes | Yes | Critically low quality review |
| Kewcharoen, 2020 | Yes | No | Yes | Partial Yes | Yes | Yes | Partial Yes | Yes | Yes | No | Yes | No | No | No | Yes | Yes | Critically low quality review |
| Chen, 2020 | Yes | No | Yes | Partial Yes | Yes | Yes | Partial Yes | Yes | Yes | No | Yes | No | Yes | Yes | No | Yes | Critically low quality review |
| Jiang, 2019 | Yes | No | Yes | Yes | Yes | Yes | Partial Yes | Yes | Yes | No | Yes | Yes | Yes | Yes | Yes | Yes | Low quality review |

Q1: **Did the research questions and inclusion criteria for the review include the components of PICO? Q2: Did the report of the review contain an explicit statement that the review methods were established prior to the conduct of the review and did the report justify any significant deviations from the protocol? Q3: Did the review authors explain their selection of the study designs for inclusion in the review?** Q4: **Did the review authors use a comprehensive literature search strategy?** Q5: Did the review authors perform study selection in duplicate? Q6: Did the review authors perform data extraction in duplicate? **Q7: Did the review authors provide a list of excluded studies and justify the exclusions?** Q**8: Did the review authors describe the included studies in adequate detail?** Q**9: Did the review authors use a satisfactory technique for assessing the risk of bias (RoB) in individual studies that were included in the review?** Q**10: Did the review authors report on the sources of funding for the studies included in the review?** Q**11: If meta-analysis was performed did the review authors use appropriate methods for statistical combination of results?** Q**12: If meta-analysis was performed, did the review authors assess the potential impact of RoB in individual studies on the results of the meta-analysis or other evidence synthesis?** Q**13: Did the review authors account for RoB in individual studies when interpreting/ discussing the results of the review?** Q14: Did the review authors provide a satisfactory explanation for, and discussion of, any heterogeneity observed in the results of the review**?Q15: If they performed quantitative synthesis did the review authors carry out an adequate investigation of publication bias (small study bias) and discuss its likely impact on the results of the review?** Q**16: Did the review authors report any potential sources of conflict of interest, including any funding they received for conducting the review?**
